# 肺癌分期分类相关的解剖学、生物学及理念

**DOI:** 10.3779/j.issn.1009-3419.2010.01.01

**Published:** 2010-01-20

**Authors:** Frank C. DETTERBECK, Lynn T. TANOUE, Daniel J. BOFFA, 永波 杨, 志刚 李

**Affiliations:** 1 Thoracic Oncology Program, Yale Cancer Center; 2 Division of Thoracic Surgery Department of Surgery, Yale University; 3 Division of Pulmonary and Critical Care Medicine, Department of Medicine, Yale University, New Haven, Connecticut; 4 天津医科大学总医院，天津市肺癌研究所，天津市肺癌转移与肿瘤微环境重点实验室

**Keywords:** 肺癌, 解剖学, 生物学, 概念, 分期

## Abstract

尽管用于此修订本的大样本量患者数据库已极大地拓宽了我们的知识面，但最新提出的肺癌分期系统仍以解剖学特征为基础。可以预见，由于所鉴定出的患者亚群数目不断增加，肺癌分期系统变得愈加复杂。表述这些亚组的临床特征有可能为我们提供肿瘤亚组特殊的生物学行为特性的线索。本文探索了可用于以解剖学为基础的新分期系统的肿瘤生物学相关观念。

分期分类系统为患有特定类型肿瘤的患者提供一种通用命名法。通用语言便于各中心间的交流，并使不同来源的观察资料得以整合，从而增进我们的共识。伴随知识的获取，人们可制定更为详尽的分期系统，因而分期系统需要定期的修改和完善。在肺癌领域，国际肺癌研究协会分期委员会担任的主要工作是发布国际抗癌联盟和国际癌症联合会（IASLC）关于癌症分期系统的修订本^[1-5]^，包括大量资料的收集和科学分析^[1-5]^。这促使我们反思分期系统的目的、我们对肺癌生物学即有认识的局限性，以及基本理念如何促进或阻碍我们做出新的判断。

建立分期系统命名法本身可界定同质患者群。由此引发的问题是如何界定同质性。虽然有许多方法可供参考，但预后是最常用的方法。事实上，这是IASLC分期委员会用作分析和分期建议的主要终点^[2, 6, 7]^。人们对分期系统的另一共同期待是其可界定适宜采用相同治疗方案的患者群。

但是，我们必须认识到预后和治疗方案并不是一成不变的，因此要不断探索可带来更好预后的治疗方法。此外，除治疗外，其它方面的进展也导致预后不断变化。影像学的发展通过最终分期的变化而影响分期组群的预后^[8, 9]^。检测方法的改进（比如CT筛查）通过改变疾病谱也影响预后^[10]^。

因此，仅根据预后对患者进行分组会带来一定的风险，即：尽管有些患者病况完全不同，但由于当时他们恰巧具有相同的预后而被分至同一组。例如，IASLC分期修订本Ⅲa期中将N2淋巴结受累的患者、T4N0M0肿瘤患者和同侧不同肺叶有多发癌结节的患者混为一组^[2]^。随着新的治疗手段不断增多，我们会发现针对同一分期组或T、N、M描述组亚群患者的最佳疗法和最终预后可能不同。

即便治疗手段、分期过程和预后不同，但根据肿瘤生物学特征进行分组的患者仍具有很多的相似性，因此理想的分期系统应反映肿瘤的生物学特性。这提示根据分子生物学特征分类比根据解剖学特征分类更实用。但是，目前我们预测肿瘤生物学特性的能力有限，而且对肿瘤的哪些生物学特征是真正的关键因素知之甚少^[11-13]^。对IASLC分期委员会来说，没有其它选择，只有采用解剖学特征来描述患者群。不过，将来我们应该采用当即可用的所有方法来界定肿瘤的生物学行为。本文根据临床表现形式提出了一种肺癌分类方法，此分类方法被认为可反映肿瘤的生物学行为。其优势在于可与IASLC分期系统所界定的解剖学描述联合使用，总结见表 1，2。

**1:** IASLC分期的TNM描述定义^*a*^

TNM	描述	亚组^*b*^
T（原发肿瘤）		
T0	无原发肿瘤	
T1	肿瘤直径≤3 cm^*c*^，被肺或脏层胸膜包绕，未累及叶支气管近端以上位置	
T1a	肿瘤直径≤2 cm^*c*^	T1a
T1b	肿瘤直径 > 2 cm但≤3 cm^*c*^	T1b
T2	肿瘤直径 > 3 cm但≤7 cm^*c*^，或肿瘤具有以下任一项^*d*^：	
	侵犯脏层胸膜，累及主支气管、距隆突≥2 cm，	
	肺不张/阻塞性肺炎蔓延至肺门但未累及全肺	
T2a	肿瘤直径 > 3 cm但≤5 cm^*c*^	T2a
T2b	肿瘤直径 > 5 cm但≤7 cm^*c*^	T2b
T3	肿瘤直径 > 7 cm^*c*^	T3 _> 7_
	或直接侵犯胸壁、横隔、膈神经、纵膈胸膜、壁层心包，	T3_Inv_
	或肿瘤位于主支气管但距离隆突 < 2 cm^*e*^，	T3_Centr_
	或全肺不张/阻塞性肺炎，	T3_Centr_
	或同一肺叶内多个肿瘤结节	T3_Satell_
T4	任何大小的肿瘤侵犯至心脏、大血管、气管/喉返神经、食管、椎体、隆突^*e*^；	T4_Inv_
	或同侧不同肺叶出现卫星结节	T4_Ipsi Nod_
N（区域淋巴结）		
N0	无区域淋巴结转移	
N1	同侧支气管和/或肺门淋巴结及肺内淋巴结的转移，包括直接蔓延累及	
N2	同侧纵隔和/或隆突下淋巴结的转移	
N3	对侧纵隔淋巴结、对侧肺门淋巴结、同侧或对侧斜角肌淋巴结或锁骨上淋巴结的转移	
M（远处转移）		
M0	无远处转移	
M1a	对侧肺叶出现卫星结节；	M1a_Contr Nod_
	或肿瘤伴有胸膜结节或恶性胸膜腔播散^*f*^	M1a_Pl Dissem_
M1b	远处转移	M1b
特殊情况：		
TX，NX，MX	无法评估T、N、M状态	
Tis	原位癌	Tis
T1^*e*^	任何大小的浅表播散型肿瘤，但限于气管壁或主支气管壁	T1_SS_
^*a*^反映了IASLC分期委员会的建议，不一定是UICC的第7版分期系统。 ^*b*^这些亚组并没有在IASLC文献中被定义，加在此处是为了方便讨论。Goldstraw *et al*., J Thorac Oncol 2007;2:706-714; Rami-Porta *et al*., J Thorac Oncol 2007;2:593- 602; Postmus *et al*., J Thorac Oncol 2007;2:686-693; Rusch *et al*., J Thorac Oncol 2007;2:603- 612. ^*c*^最大直径。 ^*d*^直径≤5 cm且具有这些特征的T2期肿瘤归为T2a。 ^*e*^中央气道内罕见的浅表播散型肿瘤归为T1。 ^*f*^如果胸腔积液细胞学检测为阴性、非血性及漏出液，则临床判断为非肿瘤性胸腔积液。 IASLC，国际肺癌研究协会；TNM，肿瘤，淋巴结，转移。 编者注：已通过UICC评议。 注：本表得到版权所有者© 2009 by the International Association for the Study of Lung Cancer复制许可		

**2:** IASLC分期分类中的分期分组^*a*^

分期分组	T	N	M
Ⅰ			
Ⅰa	T1a, b	N0	M0
Ⅰ b	T2a	N0	M0
Ⅱ			
Ⅱa	T1a, b	N1	M0
	T2a	N1	M0
	T2b	N0	M0
Ⅱb			
	T2b	N1	M0
	T3	N0	M0
Ⅲ			
Ⅲa	T1–3	N2	M0
	T3	N1	M0
	T4	N0, 1	M0
Ⅲb			
	T4	N2	M0
	T1–4	N3	M0
Ⅳ	T_Any_	N_Any_	M1a, b
^*a*^反映了IASLC分期委员会的建议，不一定是UICC的第七版分期系统。 IASLC，国际肺癌研究协会；TNM，肿瘤，淋巴结，转移。 编者注：已通过UICC评议。 注：本表得到版权所有者© 2009 by the International Association for the Study of Lung Cancer复制许可			

## 根据生物学行为分类

由于我们的了解有限，因此试图根据生物学行为来描述肿瘤特征本身即具有推测性。我们假定肿瘤有四种类型的生物学行为：临近局部侵袭型、区域淋巴结扩散型、肺内发生其它癌转移灶和全身播散型（表 3）。这些分类符合常见的临床表现形式。比如，有的患者原发肿瘤很大但没有转移，有的肿瘤很小但有淋巴结和/或远处转移，有的肺部有多处癌灶而无任何肺外受累表现。在表 4中，新的IASLC分期组群以及原发肿瘤、淋巴结和转移描述按照临床类型进行排列。

**3:** 肺癌生物学行为类型

肿瘤特征为
局部生长/侵袭型
淋巴结受累型
肺实质的多发病灶病型
全身播散型
注：本表得到版权所有者© 2009 by the International Association for the Study of Lung Cancer复制许可

**4:** 根据肿瘤生物学行为类型进行排列的IASLC分期分组^*a*^

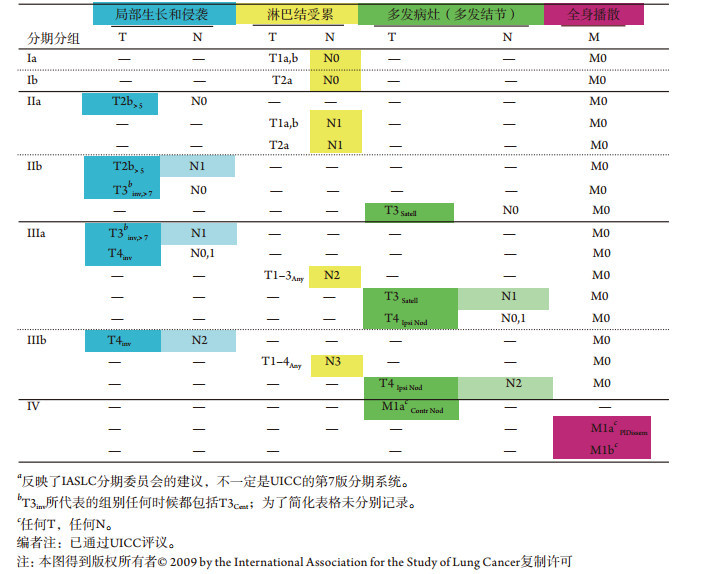

根据临床表现进行分类可能是一种重要理念，有助于我们识别同一分期组群中患者的差异性，以及不同分期组群中患者的相似性。表 4中，临床表现形式（及生物学行为的假设形式）垂直排列，分期组群水平排列。这突出了有些分期组群包含了临床表现不同的患者，以及隶属不同分期组群的亚组患者间的临床表现相似。

与辨别临床表现相比，预测肿瘤生物学行为充满了不确定性。然而，利用所观察到的个别肿瘤的临床表现来评估未来行为似乎很合理。早期肿瘤可能由于生长或发育不充分而不足以形成预测的基础。当肿瘤发育至后期，我们认为所观察到的主要表现形式可以较好地预测其未来行为。解剖学描述仍是肺癌研究最多的层面，而且是唯一拥有大样本量患者资料的特征。另外，表 4所列的分类具有其优势：即可以在全球范围应用（无需基因组分析）。因此，此种分类是一个实用的工具，便于我们研究如何预测生物学行为。

已有充足的临床经验表明一些肿瘤主要呈现局部生长和侵袭，而淋巴结或全身播散性有所减弱。许多报告证实伴有其它组织局部侵犯的患者术后生存期较好，尤其当完全切除后。单独局部治疗的预后较好，这验证了肿瘤播散性减弱的假说。常见的例子是肿瘤侵及胸壁（T3N0M0期肿瘤行R0切除后5年生存率保持在50-60%）^[14]^。有报道指出侵及脊柱^[15-17]^或隆突^[18-21]^的T4_inv_N0M0期肿瘤被完全切除后患者生存期较好。但是，由于淋巴结受累和非完全切除的患者也包含在内，因此许多数据难以被解释。在IASLC分期项目的庞大数据库中，明确记载了局部侵犯或原发肿瘤较大但无明显淋巴结受累的患者生存期较好。总的来说，虽然对局部侵润肿瘤实施有效的局部治疗仍有一定困难，但有资料支持亚组肿瘤的淋巴结或全身转移特性降低的假说。

淋巴结受累的肿瘤患者的局部和全身复发的可能性更高。更为积极的局部治疗和系统治疗越来越多。这包括Ⅱ期（N1）和Ⅲa（N2）期肿瘤完全切除后的辅助化疗、Ⅲa（N2）期肿瘤除化疗和放疗外手术作用的探讨，及对Ⅲ期（N2, 3）肿瘤行大剂量三维适形放疗和化疗^[22]^。

对肺部伴有多发肿瘤结节的患者进行分类的差异最大且最难以准确界定。人们一贯认为同一肺叶内有卫星灶的癌症患者预后较好，与无卫星灶的同种肿瘤的预后差别甚微^[23-32]^。同样，多项研究发现同侧不同肺叶有多发结节的肿瘤患者的预后相对较好，明显比存在其它形式远处转移的患者的预后好^[23, 25-30, 32, 33]^。通常这些患者肺部伴有多发性恶性结节^[27, 28, 33, 34]^。此过程极端的例子可能是那些被称为“肺炎型”腺癌的患者，其部分肺间质弥漫性受累^[35-37]^。这种“播散”发生的具体机制尚不明确，但是临床观察也似乎不符合淋巴或者血行播散的观点。有关肿瘤行为的较好观点为：肿瘤并非从一个区域“播散”至另一区域，而是形成多病灶肿瘤。这可能是由于“区域肿瘤化”，或者是由于为适应肿瘤发展模式分期，宿主微环境原因不明的改变。这些肿瘤淋巴结受累或远处播散的特性较弱^[29, 33, 37-40]^。

纳入IASLC数据库的患者比例资料（表 5）显示大多数患者（57%）通过界定淋巴结受累程度而归为一类。小部分患者（28%）以原发肿瘤生长程度为特征。以肿瘤多发结节为分期特征的患者仅占一小部分（2.5%）。虽然界定这些患者分类的实际发病率需要采集人群注册资料，但IASLC数据库提供了粗略的估计比例。此粗略评估指出IASLC分期系统的复杂性主要由患者亚组数目过少所致。

**5:** 根据生物学行为类型的IASLC分期分组相对比例^*a*^

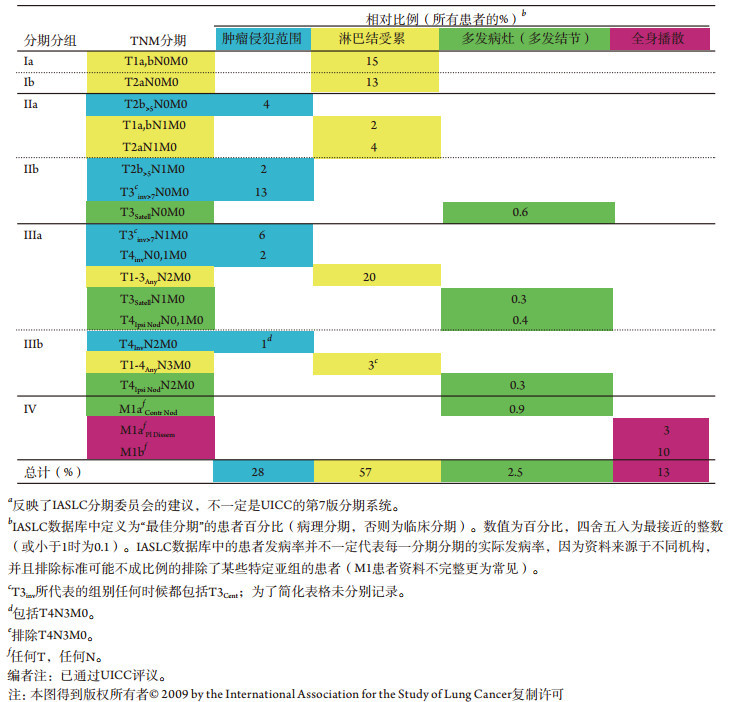

## 讨论

由于我们治疗患者、进行试验都是在先前的理念下进行的（通常缺乏资料），因此对已被接受的临床观点进行严格验证至关重要。从临床表现的形式角度来解读IASLC亚组分期具有潜在的益处。我们有理由相信肿瘤的临床表现形式与其生物学行为密切相关。这种分类可能对局部和系统治疗与具体的治疗前分期的相关性有意义。结果证实，即便表 4内纵向分类的患者处于不同分期组群中，根据其生物学行为形式采取特定治疗策略是有价值的。因为我们只关注作为整体的横向分组，所以当我们分析新治疗结果时应该考虑到这些概念，以确保不会忽略一些观察结果。

什么是肿瘤“生物学行为”的具体含义呢？从临床角度来讲，相关观点认为是肿瘤生长的速度和播散至邻近器官或远处的形式。依照定义，肿瘤具有以非限性方式生长、侵犯其它正常组织、和/或远离原发肿瘤部位进行转移和生长的能力。肿瘤的侵袭性和播散形式对预后和常规治疗手段影响最大。在细胞水平上，生物学行为包括决定细胞生长的遗传、调节和代谢因素，还总体包括肿瘤细胞、局部组织微环境和宿主间的相互作用。

我们不可避免地关注了肿瘤生物学的宏观方面，而不是细胞信号或基因组方面。显然，在阐明肿瘤生长的分子机制方面正取得巨大进展^[41-43]^。但是目前这些见解仅限于少数患者。更重要的是，对肿瘤分子生物学特征的研究离不开复杂的实验室分析，因而，从研究理念转化为临床实践并非易事。而且，目前尚不能将细胞生物学特征迅速应用于仍基于解剖学特征的肺癌分期系统。这并不意味着分子生物学特征不重要；事实上，将来在某些情况下分子生物学特征可能会成为主导因素。不过目前其尚未广泛应用于全球范围且未迅速应用于肺癌分期系统。

将生物学分类整合入分期系统的解剖学分组对Ⅲ期患者最有价值。这些患者包括占很大比例的非小细胞肺癌患者和最佳治疗方案极难制定的组别的患者，方案难以制定可能是由于Ⅲ期包含各种T和N亚组。临床表现形式有助于指导选择合适的治疗手段。例如，广泛的手术切除可治愈T4_Inv_N0M0肿瘤，但T1-3N2M0肿瘤患者获益较少。相反，新靶向药物的全身治疗对T1-3N2M0肿瘤患者有益，但对T4_Inv_N0M0肿瘤患者无益。局限性分叶切除可能对T4_Ipsi Nod_N0M0肿瘤有效，但对T1N2M0肿瘤无效。

淋巴结受累特性与远处转移特性有必要区别对待。尽管淋巴结分期的增高与远处转移发生率的升高明显相关，但人们不再认为经由淋巴系统播散是远处转移发展的机制。临床经验确切记载了许多患者经明确的根治性切除后仍发生了远处转移，但无淋巴结受累的迹象。多项研究采用敏感的检测方法发现，肿瘤细胞常见于骨髓或外周血中，即便是早期的肺癌患者亦是如此^[44-49]^。虽然检测到这些细胞与远处复发率的升高显著相关，但与淋巴结分期无关^[44-48]^。肿瘤转移的发生是由肿瘤细胞群特征的复杂的交互作用、宿主微环境以及二者之间互相作用决定的^[50]^。虽然许多患者会同时呈现淋巴结受累特性和全身转移特性，但最好是将两者区别对待。

在讨论个况肿瘤（如，T1N3M0或T4_Inv_N0M0肿瘤）时，IASLC分期分组的分类法似乎是合理的，但如何区分其它亚组尚欠明确。例如，T4_Inv_N2M0（IIIb期）肿瘤主要根据原发肿瘤的侵袭性还是根据淋巴结受累程度进行分类？此例中，原发肿瘤直接侵及淋巴结与其通过淋巴途径播散至非临近淋巴结间存在差异。同理可用于T2b_>5_N1M0、T3_Inv_N1M0和T4_Inv_N1M0肿瘤，也就是说，临近淋巴结侵犯和非临近淋巴结受累之间存在差异。

对于伴有多发病灶及淋巴结受累的患者，根据其临床表现难以推测出其生物学行为。如，多发病灶是T3_Satell_N1M0或T4_Ipsi_N1M0肿瘤最突出的特点。但是，事实上T4_Ipsi_N2M0肿瘤患者更容易合并多处转移，主要表现为以多发实质性淋巴结为唯一“远处转移”部位^[23, 24]^。也就是说，事实上这些患者更符合有淋巴结受累和全身转移特性的患者，与T1-3_Any_N2M0或T_Any_N_Any_M1b患者相似。但是，采用此方法将这些患者进行重新分类与IASLC分期委员会的结果和建议不一致。而且只有一小部分肺部有多发癌灶的患者伴有N2淋巴结受累^[2, 29]^。因此，至少在支持不同观点的资料出现之前，将T4_Ipsi_N2M0的患者从其他多发病灶的患者中区分出来，并作为IIIb期的一个亚组更为合适，且与IASLC分期委员会的建议保持一致。

目前尚不明确胸膜腔播散是否属于直接侵犯、多发病灶或远处播散。胸膜腔受累的患者类型各有不同（例如，有些患者病灶局限，脏层胸膜可见多发结节但不伴胸腔积液；有些患者肿瘤体积大伴明显的淋巴结受累及恶性胸腔积液）。事实验证了该猜想：有胸膜腔播散但不伴淋巴结转移的患者术后生存期较好（5年生存率25-30%）。然而，大多数伴有胸膜腔播散的患者的原发肿瘤体积大、伴多发转移且生存期较差。因此，在我们找到一种有效的方法来细分他们之前，我们可将这类患者视为具有远处转移能力的一种类型。

界定肺癌的生物学行为的其它方法似乎还不能与临床应用紧密结合。许多报道指出，正电子发射体层成像（PET）强度与预后有关，提示PET强度可作为一种肿瘤代谢率和转移特性的指标^[52]^。但是，通常这些报道并未考虑其它预后因素，如肿瘤大小或分期。一项详细的对照、前瞻性研究发现，PET强度并不能提供有意义的独立预后信息，且肿瘤大小和分期是主导因素^[11]^。人们也十分关注肿瘤细胞的遗传学改变的特征^[53, 54]^。但是大部分研究都存在方法学方面的缺陷，且无法用于临床^[55]^。而且，作为同一结果的预测因子，不同的研究鉴定出的基因不同，且很少有重叠^[56]^。总之，目前基于遗传学特征的预后预测方法并不优于传统的基于临床和病理学因素的预后预测方法^[12, 56]^。IASLC分期委员会也考虑到了这点，并认为肿瘤的分子特征尚未得到充分研究，因此未被用于目前的分期修订。

我们认为采用临床表现形式作为预测肿瘤行为的潜在方法，是探索的合理途径。显然，是否能够推测出肿瘤进展的特定形式需要实验来决定。因为采用解剖学特征来界定生物学类似组群并不完善，该分类方法似乎无法预测所有亚组的行为类型。然而，我们相信生物学整合理念值得探讨。我们期待对这种分类进行更广泛的讨论，临床研究将有助于深入理解合理处理肺癌的肿瘤生物学。
